# Supplementary material for: An ultrathin conformable vibration-responsive electronic skin for quantitative vocal recognition
Source: Nat Commun. 2019 Jun 18;10:2468. doi: 10.1038/s41467-019-10465-w (PMC6581939; doi:10.1038/s41467-019-10465-w)
Supplement: Supplementary file 1 — Supplementary Information [file 41467_2019_10465_MOESM1_ESM.pdf]

Supplementary Information

An Ultrathin Conformable Vibration-Responsive Electronic Skin for Quantitative Voice Recognition

Lee et al.

■ Supplementary Figure List

Supplementary Figure 1. Experiment measuring the skin vibration when human speaks

Supplementary Figure 2. Schematic illustration of the overall fabrication process

Supplementary Figure 3. Configuration for 800 and 200 μm width diaphragms

Supplementary Figure 4. Details of interface conditioning circuit diagram connected to the sensor

Supplementary Figure 5. The stiffness measurement for the diaphragm with and without air holes

Supplementary Figure 6. Simulation analysis for the natural frequency of a 400 μm-width diaphragm structure

Supplementary Figure 7. Experimental setup for measuring vibration sensing performance

Supplementary Figure 8. Effect of diaphragm diameter and the number of diaphragms on the sensitivity

Supplementary Figure 9-11. Effects of the diaphragm diameter, the diaphragm thickness and the diaphragm-support thickness on the diaphragm properties

Supplementary Figure 12. Analysis on noise power spectral density of our device

Supplementary Figure 13. Signal to Noise Ratio (SNR) of the device

Supplementary Figure 14. The diaphragm deflection and the corresponding capacitance modulation

Supplementary Figure 15. Frequency spectra with wider frequency range for the composed sheet music

Supplementary Figure 16. Comparison for the acceleration magnitude of the skin vibrations

Supplementary Figure 17. The effect of shaking head back and forth on the performance of our device

Supplementary Figure 18. The effect of covering mouth on the performance of our device

Supplementary Figure 19. Voice dosimetry for a woman participant

Supplementary Figure 20. Photograph showing the neck skin after three hours of wearing our device

Supplementary Figure 21. Experimental setup for voice authentication and voice remote control system

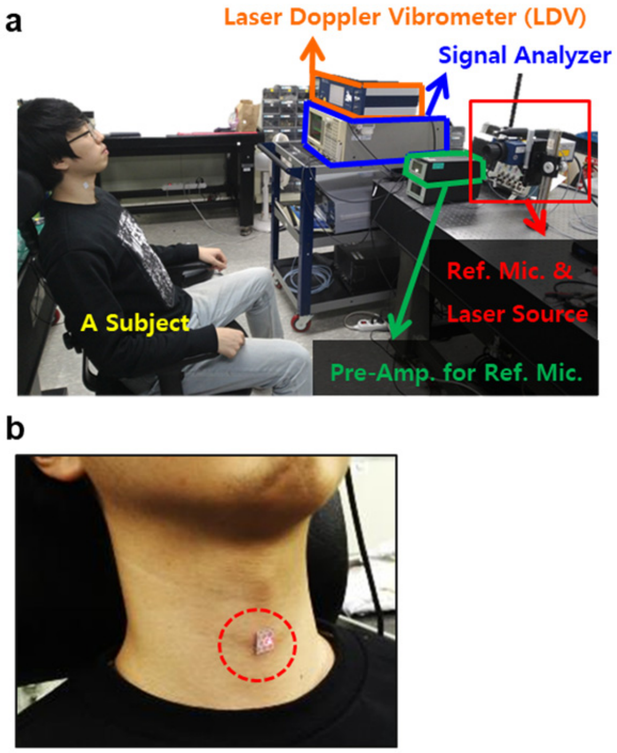

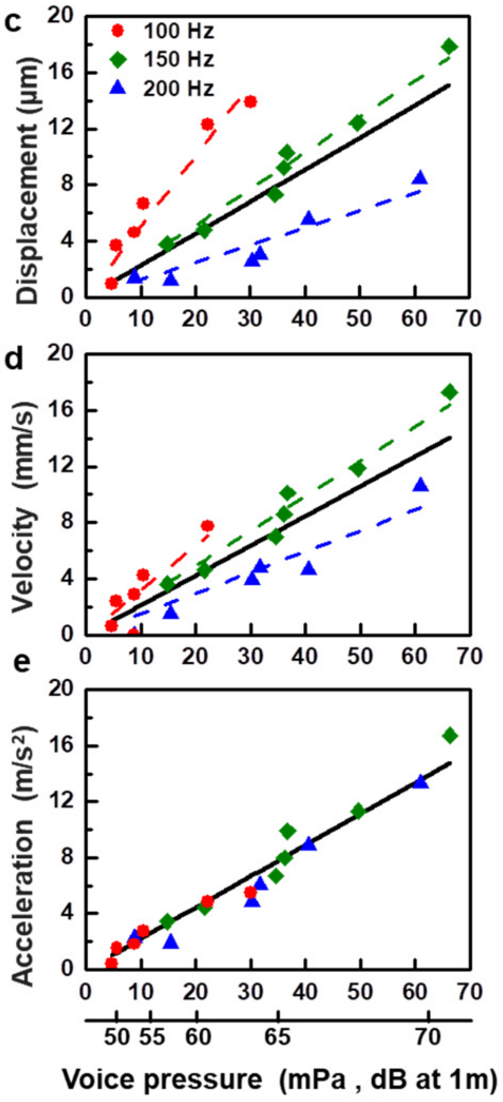

f

	Coefficient of determination (R^2) for all data
Displacement	**0.81019**
Velocity	**0.91708**
Acceleration	**0.98283**

Supplementary Figure 1. Experiment measuring the skin vibration when human speaks. a, Photographic image of experimental setup. **b,** Photographic image of laser-focused location for laser Doppler vibrometry. **c-e,** The degrees of the skin vibrations were plotted with three vibration parameters such as displacement (**c**), velocity (**d**) and acceleration (**e**), when human speaks at various voice pressure and three fundamental voice frequencies of 100 Hz (red circle), 150 Hz (green diamond) and 200 Hz (blue triangle). Each dashed line of red, green and blue in (**c,d**) is the trend line for the skin vibration values of corresponding frequencies, respectively. Black lines in (**c-e**) represent trend lines for all data in each graph. **f,** The table showing coefficients of determination for all data in each graph. The graph of (**e**) is the same with Figure 1a.

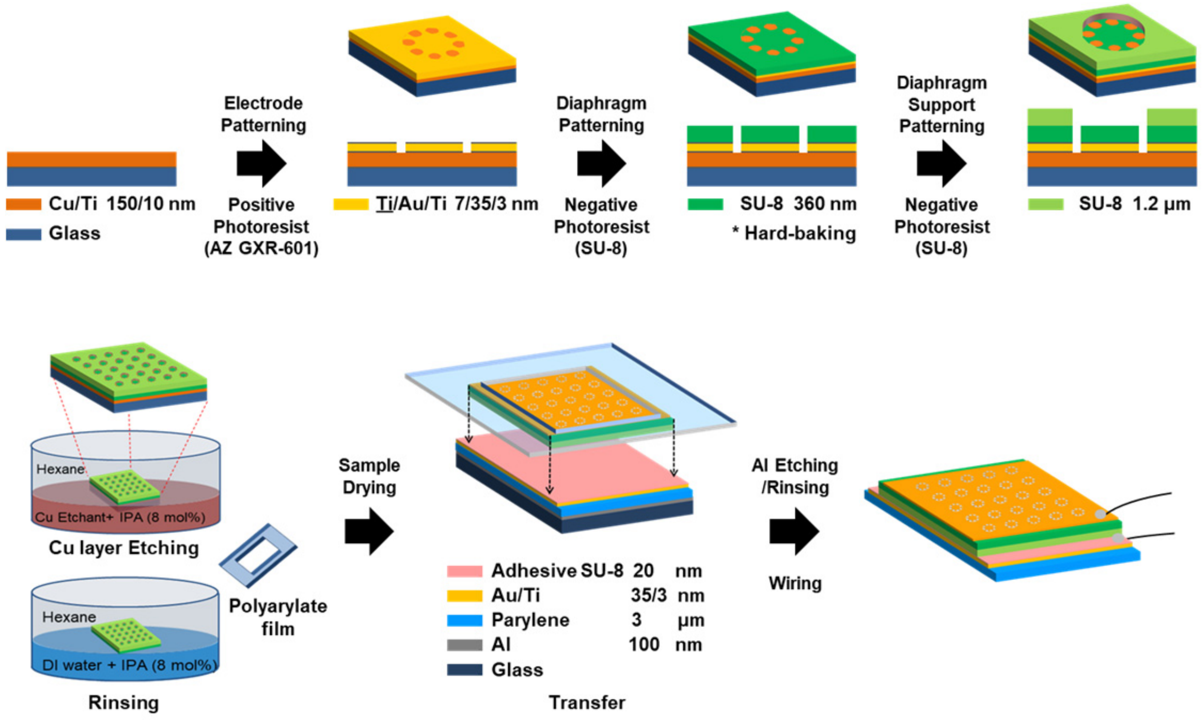

Supplementary Figure 2. Schematic illustration of the overall fabrication process

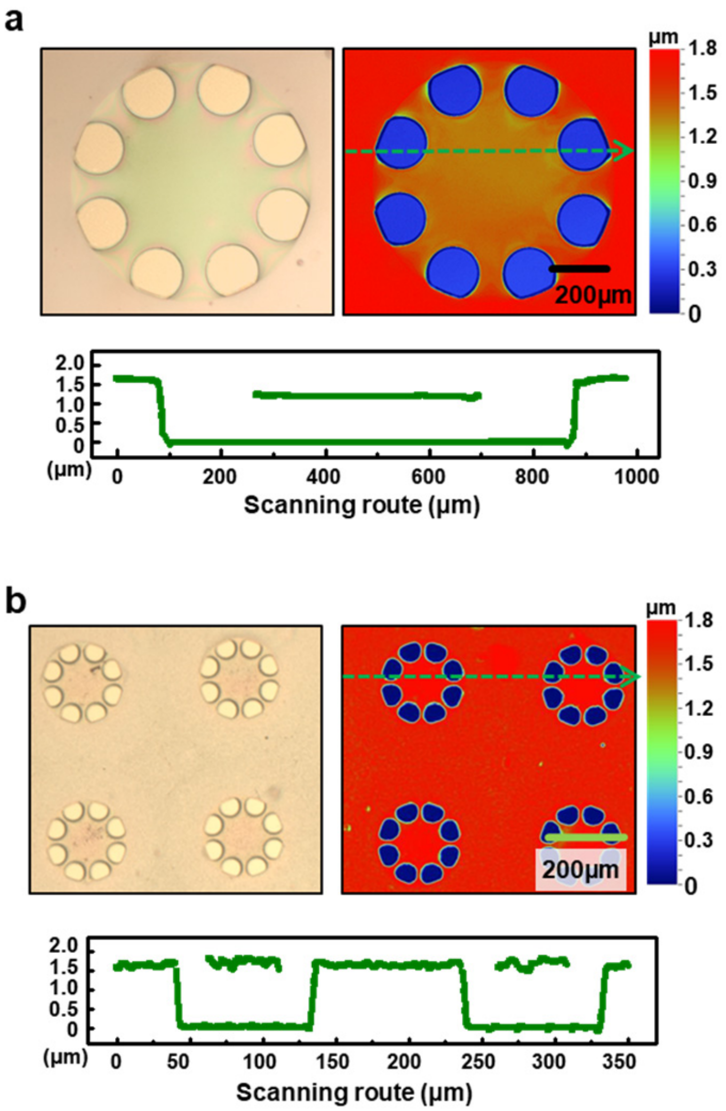

Supplementary Figure 3. Configuration for 800 and 200 µm width diaphragms. OM images, 3D profiling contours and cross-sectional profiles of the suspended diaphragms of 800 µm (**a**) and 200 µm width diameter (**b**). Scanning routes of the cross-sectional profiles are shown as the green arrow lines on 3D profiling contour data (upper right). The results show that the ultrathin (400 nm) diaphragms of 800 and 200 µm width are suspended on a 1.2 µm thick epoxy resin (SU-8) support, and their initial downward deflection are less than 100 nm.

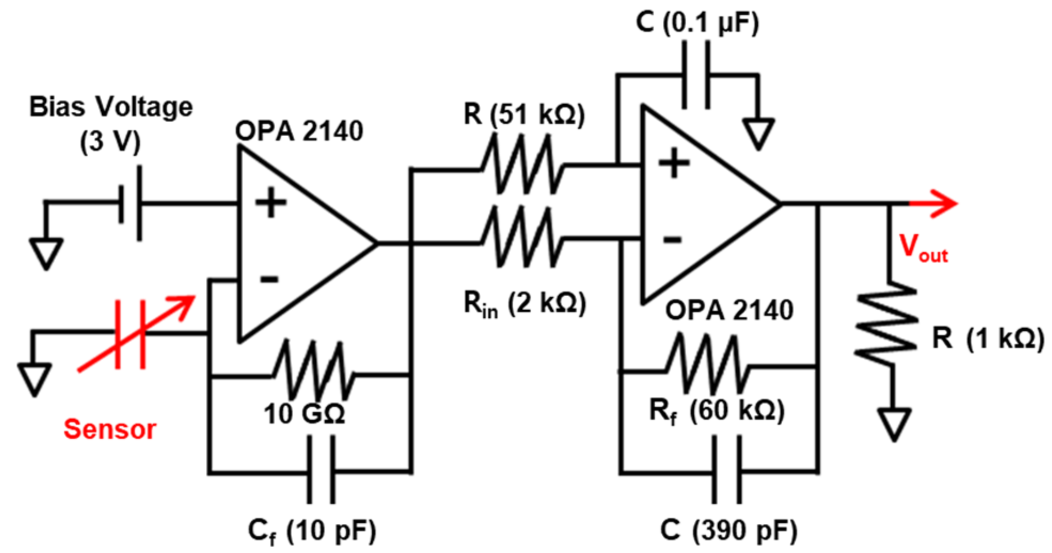

Supplementary Figure 4. Details of the interface conditioning circuit diagram connected to the sensor. The conversion ratio of modulated capacitance to output voltage is 9 V pF^{-1} by bias voltage (3 V), feedback capacitance ($C_f = 10$ pF), feedback resistance ($R_f = 60$ kΩ) and input resistance ($R_{in} = 2$ kΩ) (see fourth term of the righthand of Supplementary Equation (1)).

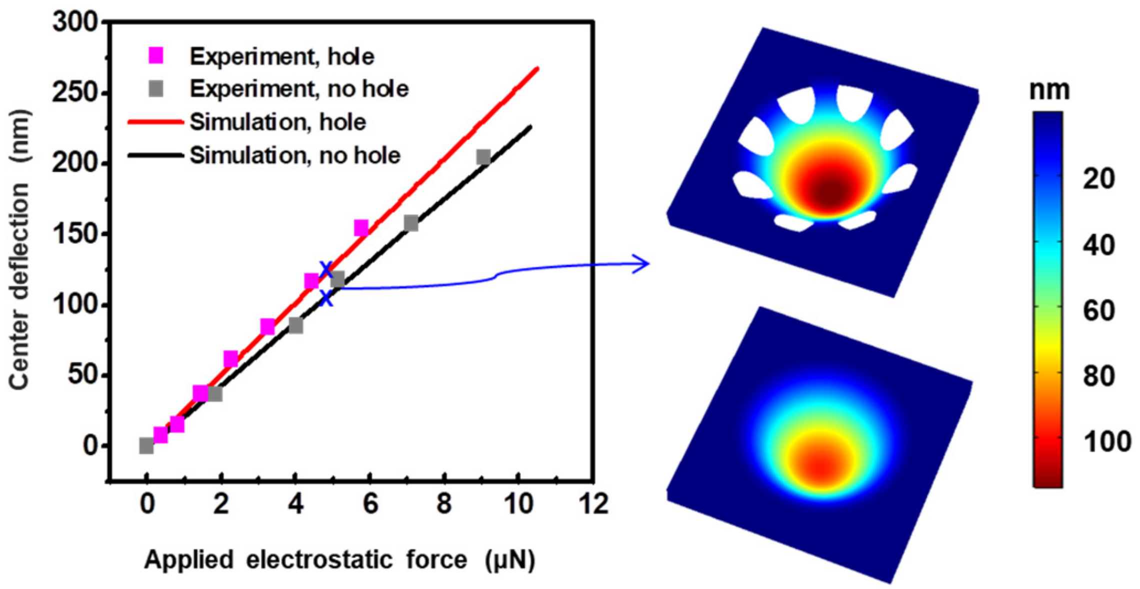

Supplementary Figure 5. The stiffness measurement for the diaphragm with and without air holes. The change of the diaphragm deflection to the applied electrostatic force was measured (Pink and gray square) and simulated (Red and black line) for the diaphragm with and without holes, respectively. The electrostatic force was applied and measured based on DC voltage bias between the upper and lower electrodes (Supplementary Note 7). The diaphragm diameter was 400 μm, the diaphragm thickness was 400 nm and the support thickness was 1.2 μm. The results show that measured values coincide with the simulation data. The stiffness was obtained by analyzing the reciprocal constant of the trend lines for measured values, based on the load-deflection model for a circular diaphragm (Supplementary Note 3). The measured stiffness for the diaphragm with holes (37.71 N m^{-1}) is 16 percent lower than the stiffness for the diaphragm without holes (44.71 N m^{-1}). The effect of the diaphragm holes on stiffness is reflected in the stiffness calibration constant ($\kappa = 0.84$) in the Supplementary Note 3. Two blue 'x's in the graph represent the 3D contours of the diaphragm deflections in the right side, which show that the diaphragm with holes is more deflected than that without holes under the same applied force due to lower stiffness of the diaphragm.

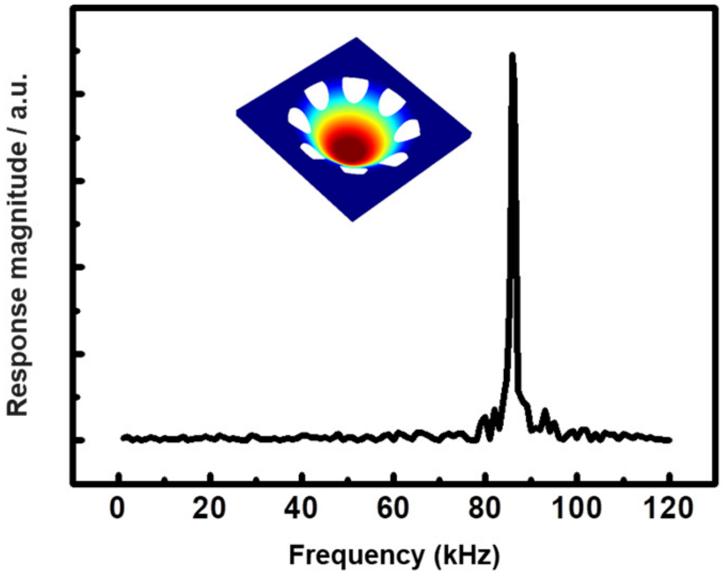

Supplementary Figure 6. Simulation analysis for the mechanical natural frequency of a 400 μm-width diaphragm structure with holes. The diaphragm diameter was 400 μm and the diaphragm thickness was 400 nm. The simulated value of the natural frequency ($f_{0,\ simul.}$) is approximately 86 kHz.

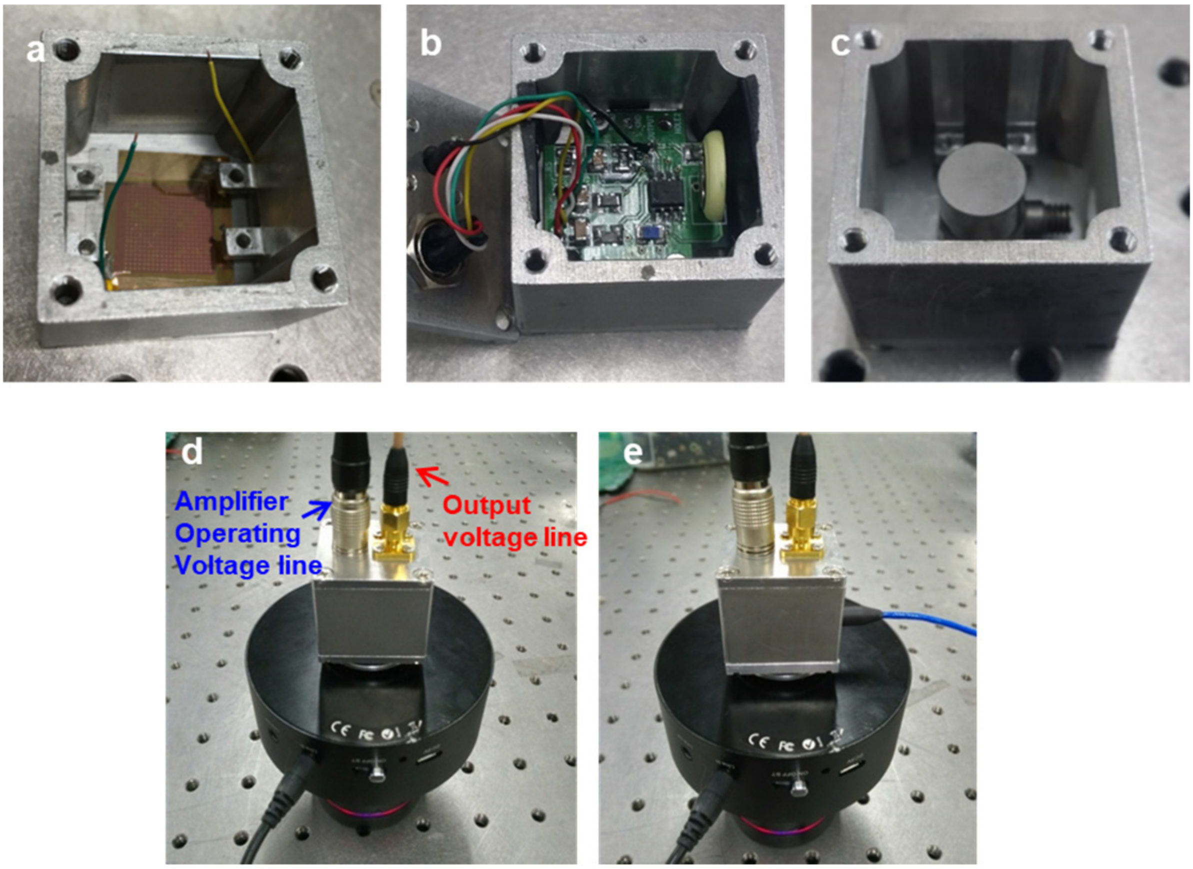

Supplementary Figure 7. Experimental setup for measuring vibration sensing performance. a-b, Photographic images of the device (**a**) and the electric circuit (**b**) located in a shielding box made of aluminum. **c,** Reference accelerometer (PCB, 352C33) was located in the same position for the mechanical balance at the comparison experiment to the device. **d,** The shield box, connected with amplifier operating voltage line and output voltage line, was placed on an electromagnetic vibration speaker. **e**, At the experiment for the reference accelerometer, the voltage lines function for only mechanical balance.

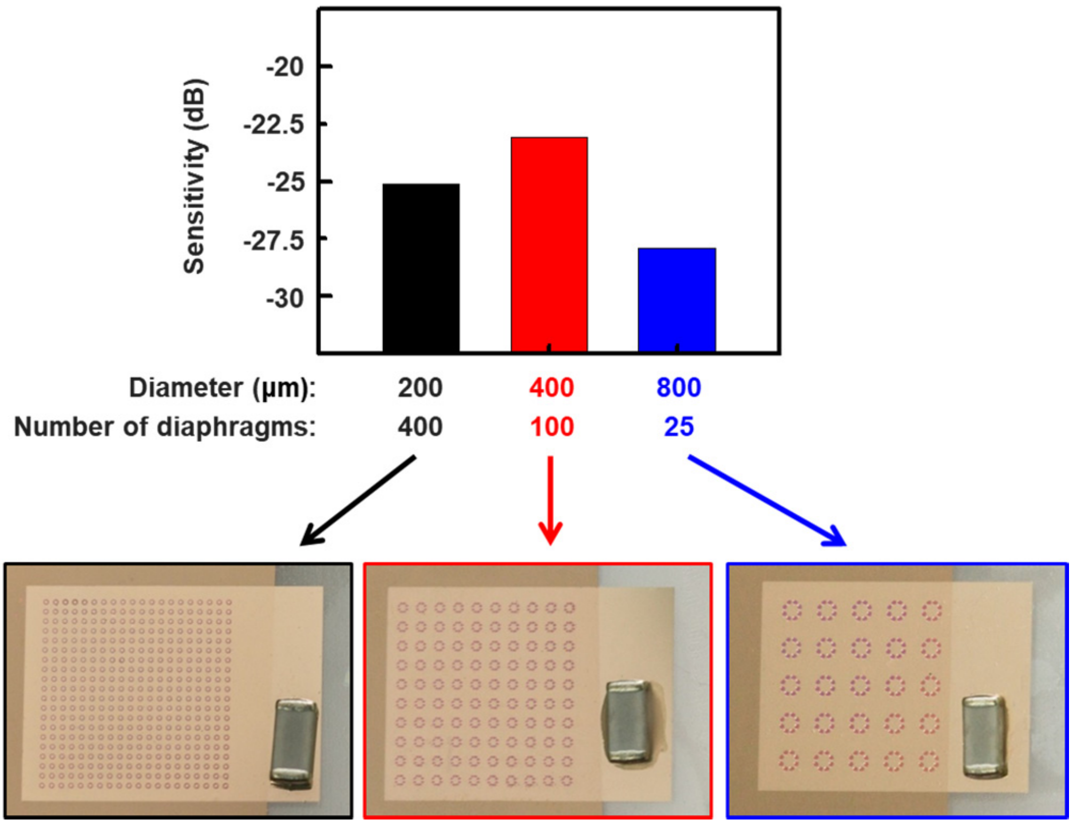

Supplementary Figure 8. **Effects of diaphragm diameter and the number of diaphragms on the sensitivity.** The output sensitivity data and photograph images of an array of 400, 100 and 25 diaphragms with 200, 400 and 800 μm diameter in the same area, respectively. The same chip capacitors (KEMET, C1206C391J1GACTU; 1.60 x 3.20 mm^2) were used as the scale bar. The images show that the maximum number of diaphragms that can be fabricated in the same area is inversely proportional to the square of the diaphragm diameter.

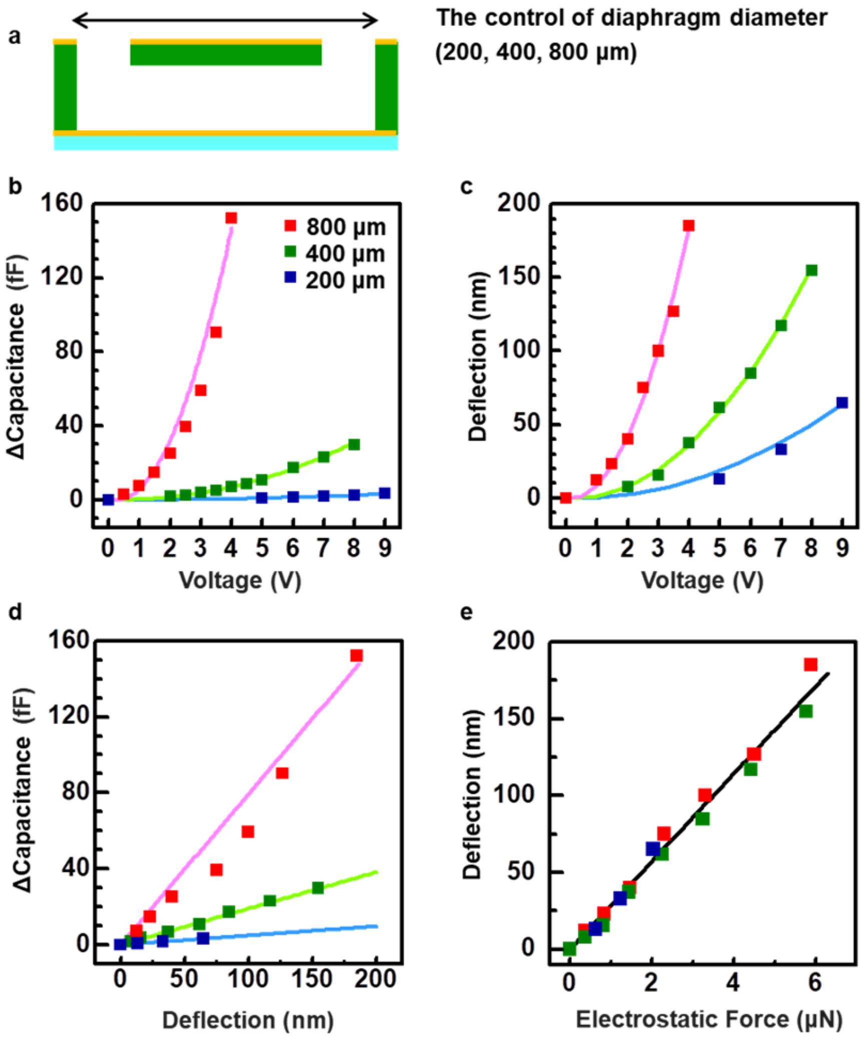

Supplementary Figure 9. Effect of the diaphragm diameter on the diaphragm properties. a-c, After controlling a suspended diaphragm diameter as 200, 400 and 800 μm (**a**), we measured and simulated the change of the capacitance (**b**) and the diaphragm deflection (**c**) under DC voltage on the upper and lower electrodes. The thickness of the diaphragm and the diaphragm support was 400 nm and 1200 nm, respectively. **d**, Then we analyzed the diaphragm deflection and the corresponding capacitance change at the same DC voltage. **e**, We extracted the stiffness of the diaphragm by plotting the diaphragm deflection and the corresponding electrostatic force applied by the DC voltage bias (details of the electrostatic force measurement are in Supplementary Note 7). For all graphs in (**b-e**), colored squares and lines represent experimental measured values and simulation data for the diaphragms with 200 μm (blue), 400 μm (green) and 800 μm (red) width diameters, respectively. Black line in (**e**) is the trend line for the measured values, which represents that the stiffness values of the diaphragms are on the same level in the cases of all diameters.

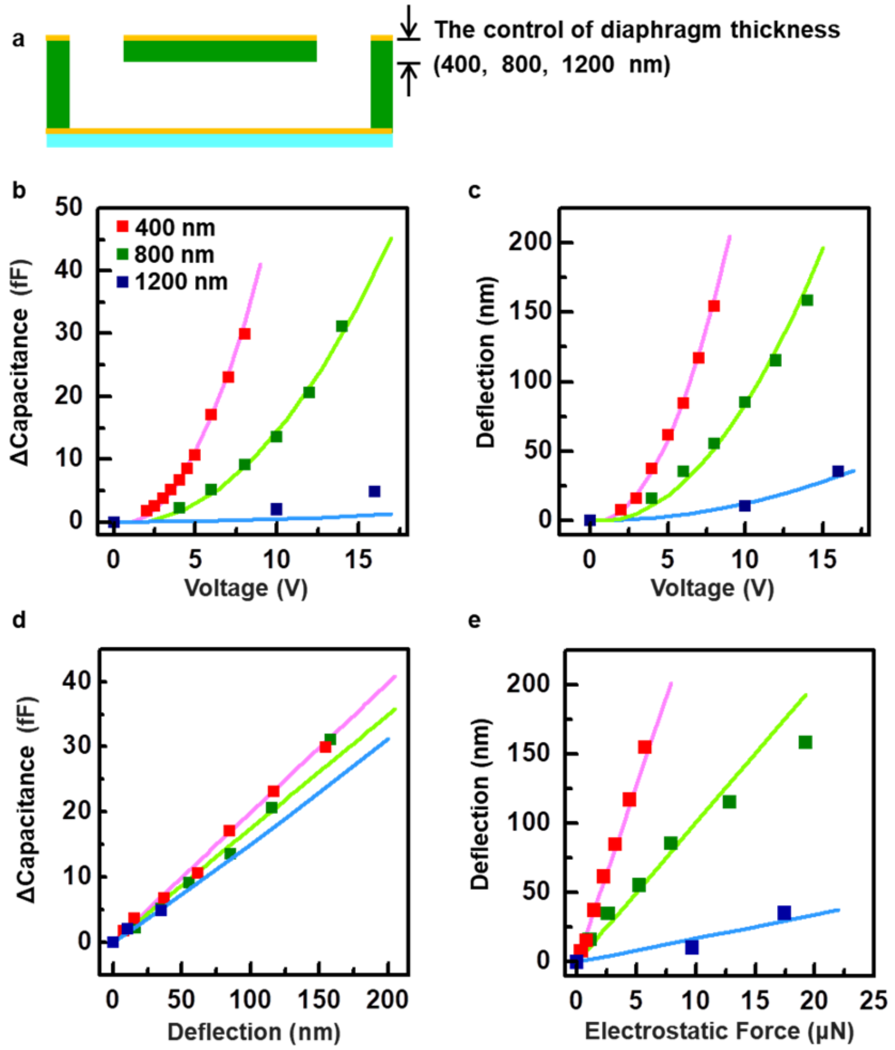

Supplementary Figure 10. Effect of the diaphragm thickness on the diaphragm properties. a-c, After controlling the diaphragm thickness as 400, 800 and 1200 nm (**a**), we measured and simulated the change of the capacitance (**b**) and the diaphragm deflection (**c**) under DC voltage on the upper and lower electrodes. The diameter of diaphragm was 400 μm and the thickness of the diaphragm support was 1200 nm. **d**, Then we analyzed the diaphragm deflection and the corresponding capacitance change at the same DC voltage. **e**, We extracted the stiffness of the diaphragm by plotting the diaphragm deflection and the corresponding electrostatic force applied by the DC voltage bias (details of the electrostatic force measurement are in Supplementary Note 7). For all graphs in (**b-e**), colored square and lines represents experimental measured value and simulation data for the diaphragm with the thickness of 400 nm (red), 800 nm (green) and 1200 nm (blue), respectively. The stiffness values can be compared by analyzing the reciprocal slope of trend lines for the measured values. The graph in (**e**) shows that the stiffness increases with increasing the diaphragm thickness.

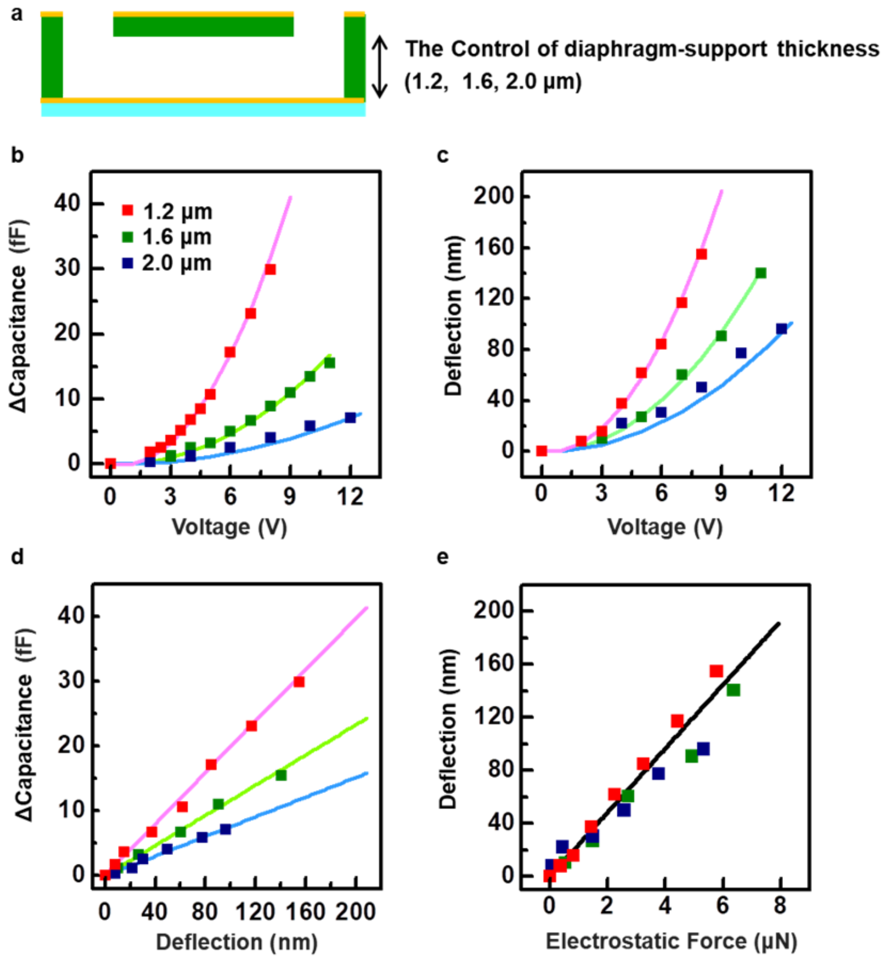

Supplementary Figure 11. Effect of the diaphragm support thickness on the diaphragm properties. a-c, After controlling the thickness of the diaphragm support as 1.2, 1.6 and 2.0 μm (**a**), we measured and simulated the change of the capacitance (**b**) and the diaphragm deflection (**c**) under DC voltage on the upper and lower electrodes. The diameter and thickness of the diaphragm were 400 μm and 400 nm, respectively. **d**, Then we analyzed the diaphragm deflection and the corresponding capacitance change at the same DC voltage. **e**, We extracted the stiffness of the diaphragm by plotting the diaphragm deflection and the corresponding electrostatic force applied by the DC voltage bias (details of electrostatic force measurement are in Supplementary Note 7). For all graphs in (**b-e**), colored square and lines represents experimental measured value and simulation data for the diaphragm support with the thickness of 1.2 μm (red), 1.6 μm (green) and 2.0 μm (blue), respectively. Black line in (**e**) is the trend line for the measured values, which represents that the stiffness values of the diaphragms are on the same level in the cases of diaphragm supports with all thickness.

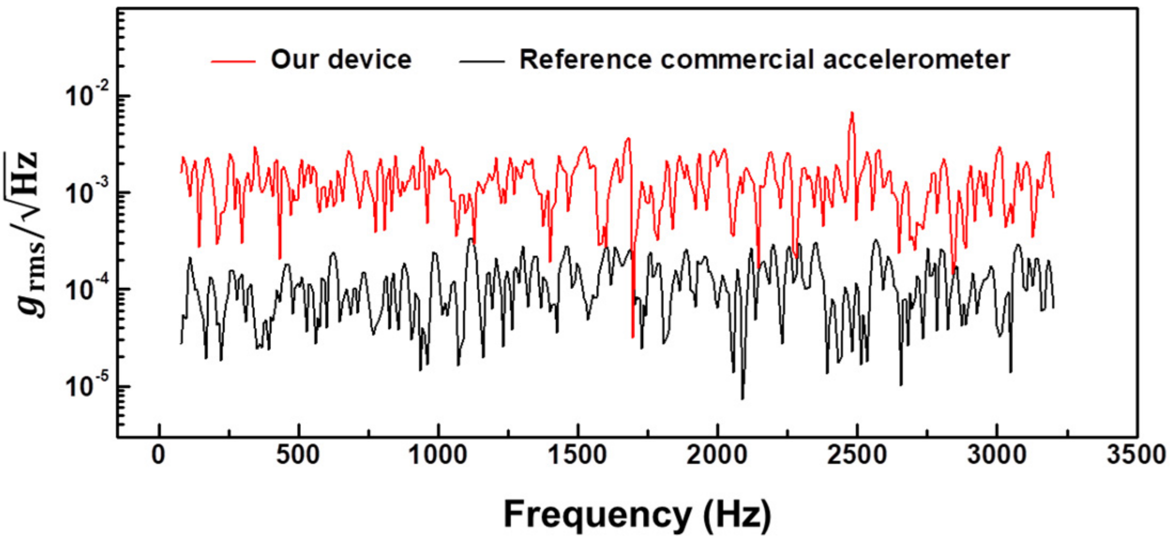

Supplementary Figure 12. Analysis on noise power spectral density of our device. The data was obtained by connecting our device and reference commercial accelerometer (PCB Piezotronics, 352C33) to the signal analyzer (Stanford Research Systems, SR785), and analyzing noise power spectral density for voice frequency range (80 ~ 3400 Hz).

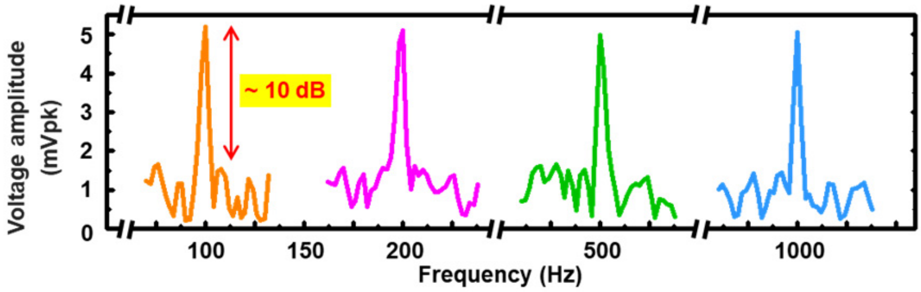

Supplementary Figure 13. Signal to Noise Ratio (SNR) of the device. The device exhibited more than 10 dB of SNR at 0.02 *g* and several frequencies (100, 200, 500 and 1000 Hz) of the base acceleration. Note that the smallest neck-skin vibration when human speaks was obtained to approximately 0.02 *g*. The device was consisted of an array of 400 hole-patterned diaphragms with low tan δ polymer, each of which had the diaphragm diameter of 400 μm, the diaphragm thickness of 400 nm and the diaphragm support thickness of 1.2 μm.

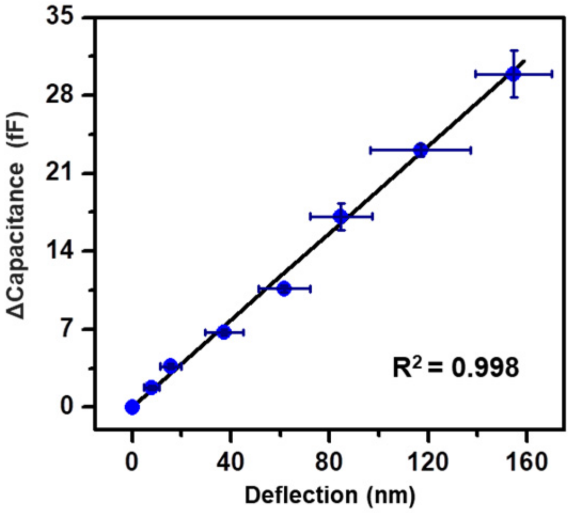

Supplementary Figure 14. Diaphragm deflection and the corresponding capacitance modulation. The capacitance change of an element of 400 μm width diameter diaphragm was measured when the diaphragm was deflected under DC voltage on the upper and lower electrodes. The diaphragm thickness was 400 nm and the diaphragm support thickness was 1.2 μm. Blue circle and black lines mean the measured and simulation result, respectively. The error bars represent the s.d. of the test results for at least three samples. The coefficient of determination (R^2) of the measured values for the trend line is 0.998, which indicates the high and linear correlation between the diaphragm deflection and the corresponding capacitance modulation.

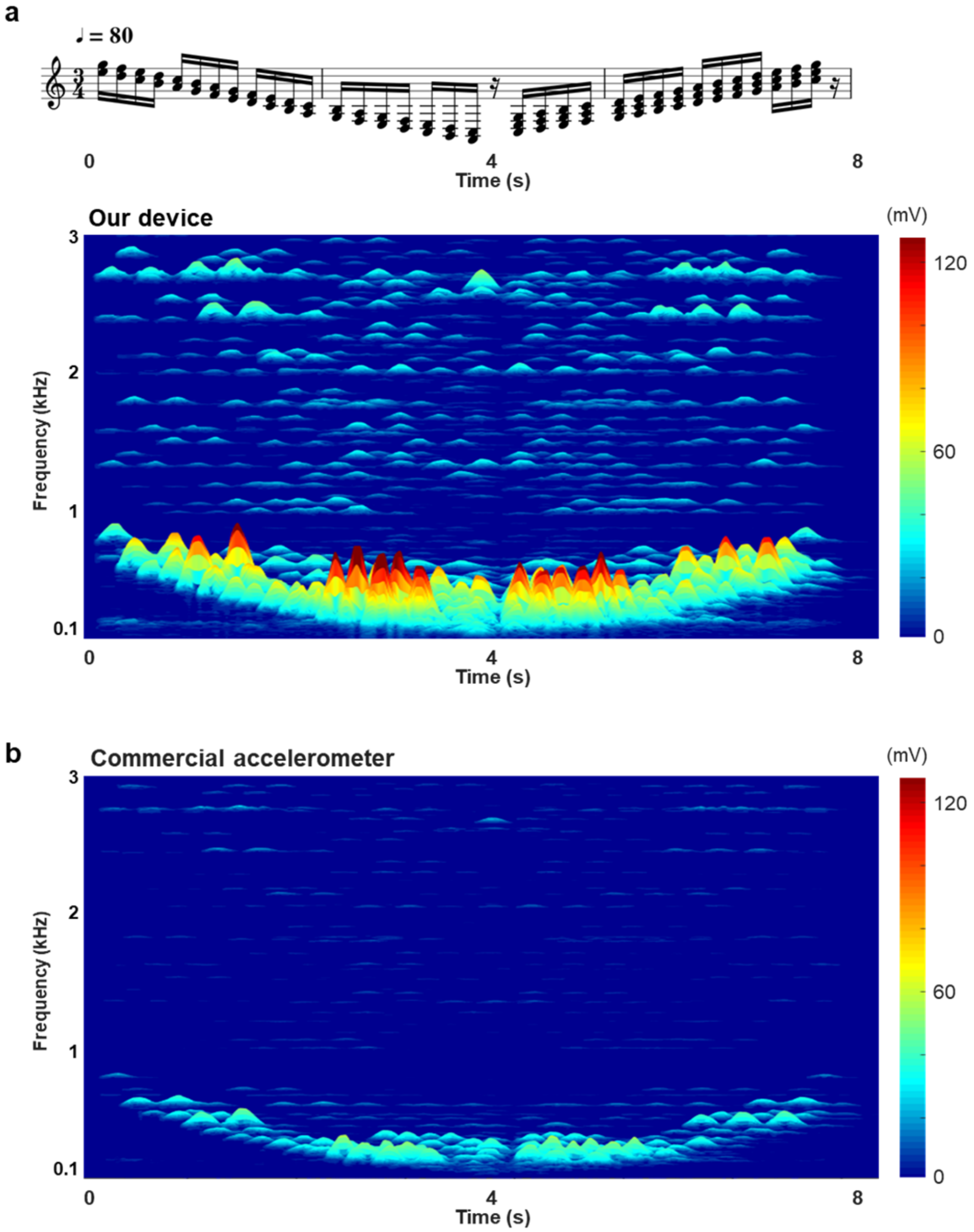

Supplementary Figure 15. Frequency spectra with wider frequency range for the composed sheet music. When the sheet music was played by a vibration speaker, frequency spectrum corresponding to the vibrational output recorded by our device (**a**) and a commercial accelerometer (sensitivity: 100 mV g^{-1} for 10 ~ 10,000 Hz) (**b**). Frequency range of frequency spectra is broader (80 Hz to 3000 Hz) than that of frequency spectra obtained by the device (Fig. 3b) and commercial accelerometer (Fig. 3c). Figure 3 shows only a few-fundamental frequencies range corresponding to sheet music (Fig. 3a) to facilitate comparison between our device and the commercial accelerometer.

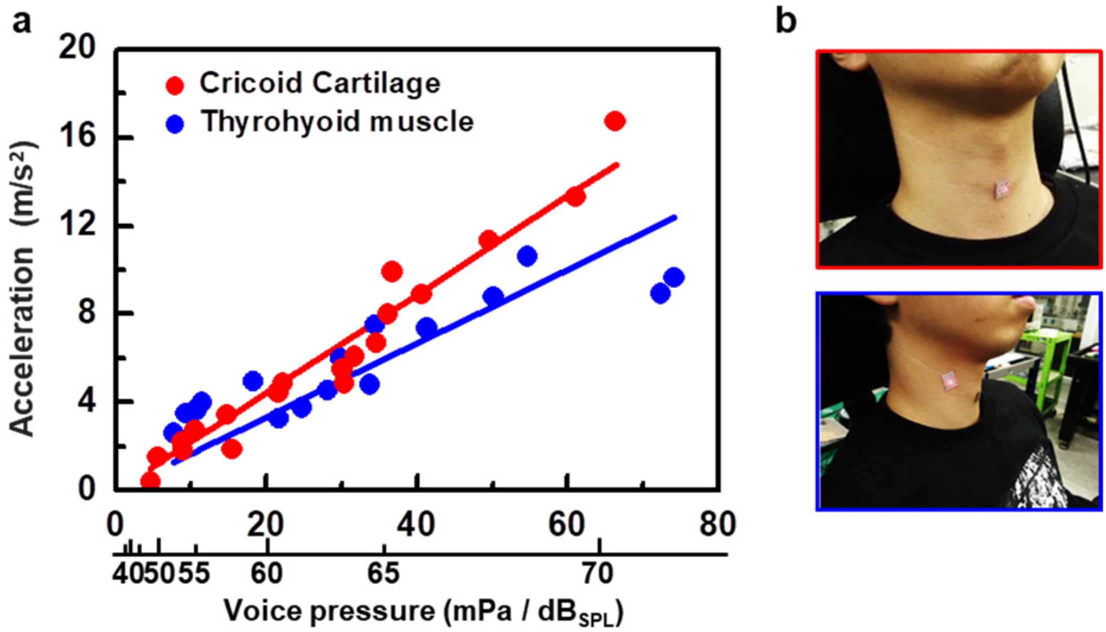

Supplementary Figure 16. Comparison for the acceleration magnitude of the skin vibrations. a, The graph for skin acceleration magnitude at two positions of cricoid cartilage (Red) and thyrohyoid muscle (Blue), when human speaks at various voice pressure with three representative fundamental voice frequency of 100, 150 and 200 Hz. **b**, Photographic images showing two measuring spots (Red: Cricoid cartilage and Blue : Thyrohyoid muscle).

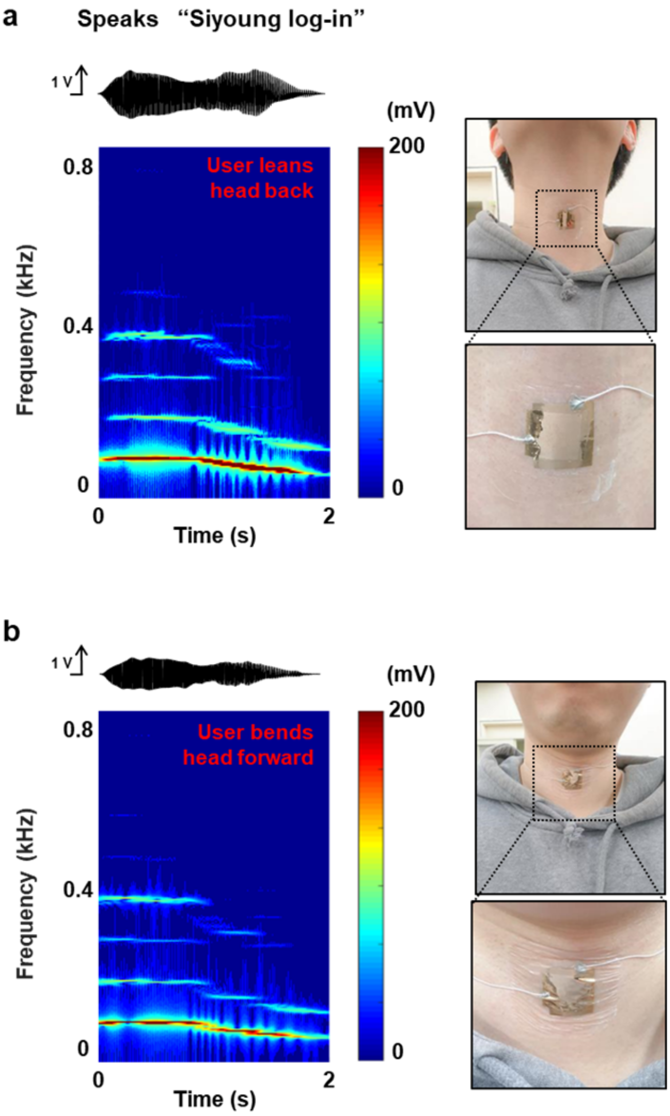

Supplementary Figure 17. The effect of shaking head back and forth on the performance of our device. Output waveform and frequency spectrum measured by our device attached on the neck skin while the user phonated "Siyoung log-in" with leaning head back (**a**) and bending head forth (**b**). Photographs in (**a**) and (**b**) show our device attached on the neck skin when the user shakes the head.

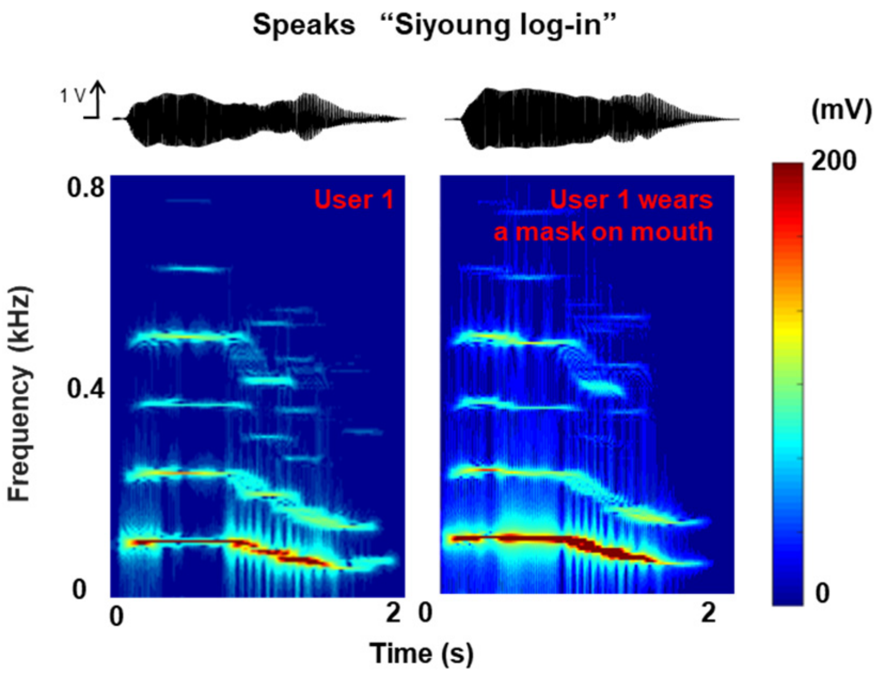

Supplementary Figure 18. The effect of covering mouth on the performance of our device. Comparison of waveforms and frequency spectra measured by our device when the same user said "Siyoung-login" without and with a mask on his mouth. The mask hardly affects the performance of our device, because the device recognizes human voice by detecting skin vibration instead of voice pressure.

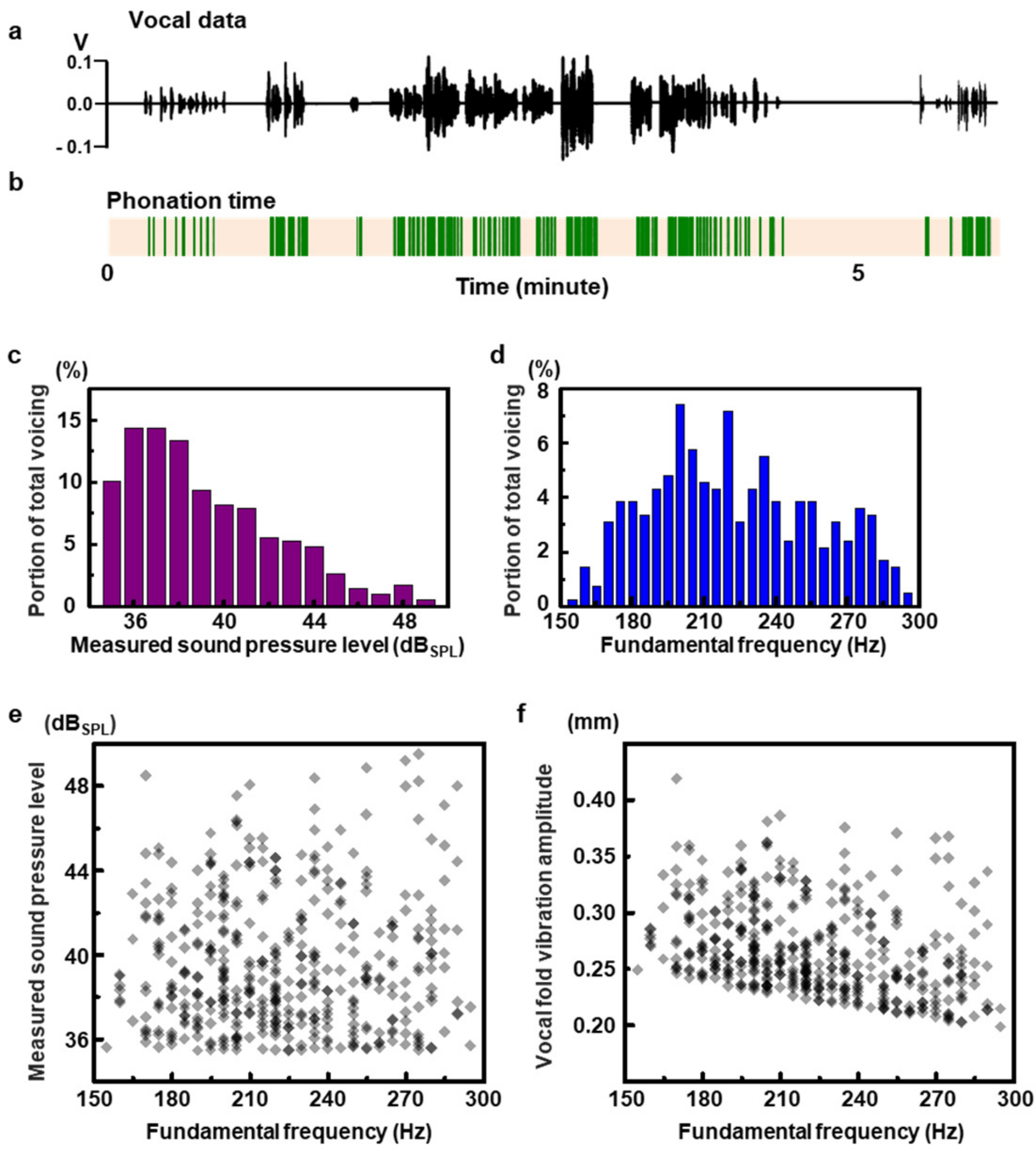

Supplementary Figure 19. Voice dosimetry for a woman participant. a, Vocal data obtained by our device. **b**, Phonation time measured by dividing the vocal data into 100-ms intervals and distinguishing between speaking and non-speaking. **c-d,** Histogram of sound pressure levels (**c**) and fundamental voice frequencies (**d**), each of which is extracted from the vocal data corresponding to speaking. **e**, The graph showing the occurrence of particular combinations of the fundamental frequencies (horizontal axis) and the sound pressure levels (vertical axis). **f**, The profile of the amplitudes of vocal fold vibrations analyzed based on vocal fold length and the measured sound pressure levels and fundamental voice frequencies (see Supplementary Equation (12)).

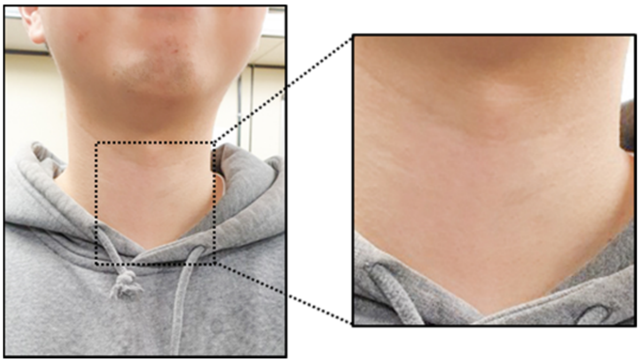

Supplementary Figure 20. Photograph showing the neck skin after three hours of wearing our device.

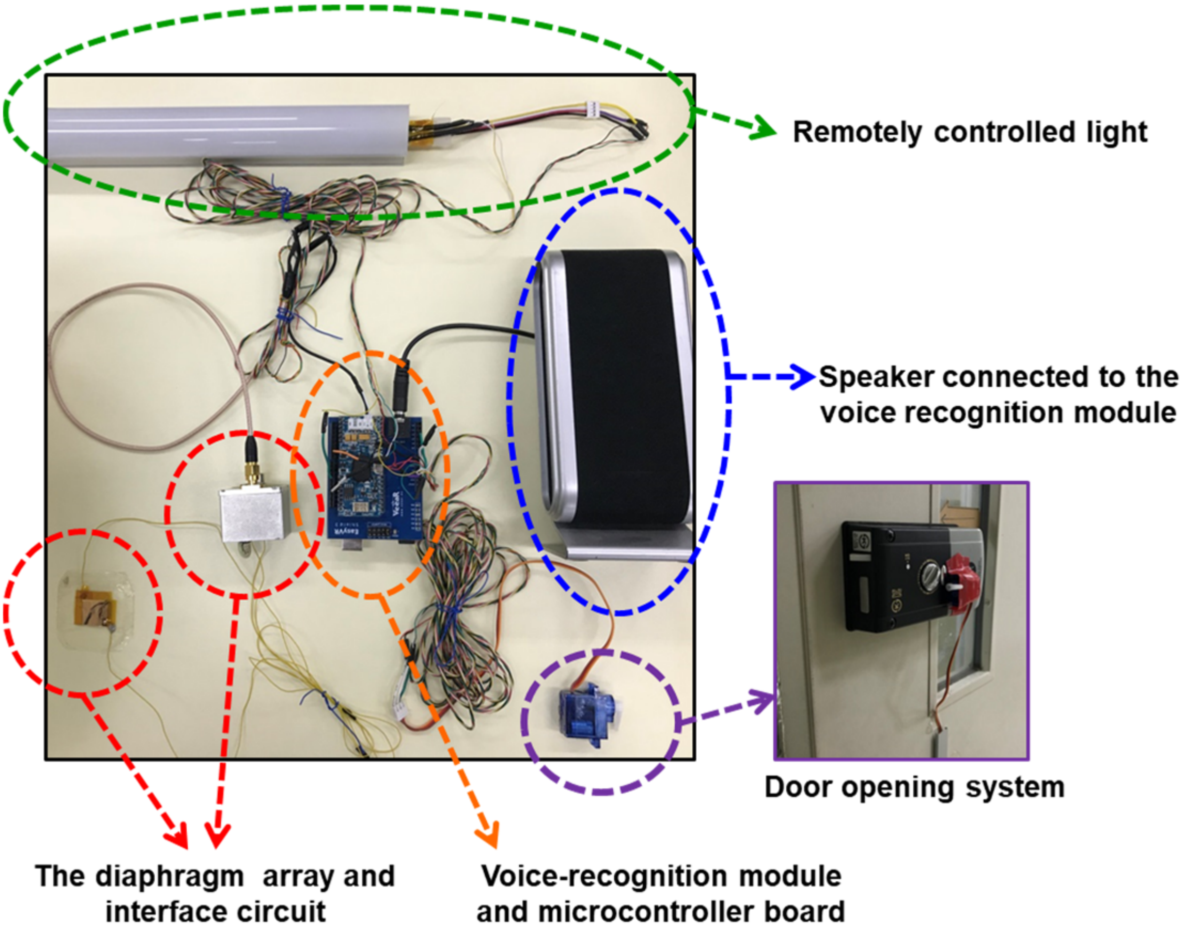

Supplementary Figure 21. Experimental setup for voice authentication and voice remote control system.

■ Supplementary Notes

Supplementary Note 1: Signal transfer function

The output voltage (V_{out}) of the device located on vibrating base is predicted as

$$V_{out} \propto a_{base} \times \frac{1}{\omega_n{}^2 \sqrt{(1-f^2)^2 + tan^2\phi_t}} \times \frac{\Delta C}{\Delta d_{cap}} \times \frac{V_{bias} \times R_f}{C_f \times R_{in}} \cdots (1),$$

where a_{base} [m s^{-2}] is the acceleration of the base vibration, ω_n [rad s^{-1}] is the natural frequency of the suspended diaphragm, f is the ratio of the input vibrational frequency to ω_n, ϕ_t is the phase lag of the diaphragm strain with respect to the applied stress, $\tan\phi_t$ represents the material and structural damping effects of the diaphragm, ΔC [F] is the capacitance change, Δd_{cap} [m] is the vibration distance between top and bottom electrodes, V_{bias} [V] is the constant voltage applied to the diaphragm, and C_f, R_f and R_{in} represent feedback capacitance, feedback resistance and input resistance of the interface circuit, respectively (Supplementary Fig. 4). The diaphragm natural frequency is equivalent to $k^{0.5}m^{-0.5}$, where k [N m^{-1}] is related to both the diaphragm stiffness and the resistive force of the air film under the diaphragm, and m [kg] is the diaphragm mass. Each term in Supplementary Equation (1) represents the sensing processes, successively. The first and second terms indicate the deflection amplitude of the diaphragm on a vibrating base[1]. The third term represents the capacitance change as a diaphragm deflects. The fourth term is a conversion ratio between the output voltage and the capacitance change.

In Supplementary Equation (1), only the second term depends on the frequency of the input acceleration; therefore, the frequency response of our device is solely affected by ω_n and $tan\phi_t$. The second and third terms consists of the sensitivity change factors: The stiffness and weight of diaphragm, the resistive force of the air film under the diaphragm and the ratio of capacitance modulation to the change of the gap under the diaphragm. These factors are affected by the structural parameters of the device: diaphragm diameter, diaphragm thickness and support thickness.

When the device is on the neck skin, a_{base} can be replaced by $22.2P_{voice}$ [g], where P_{voice} [Pa] is the voice pressure at 1 m when human speaks. The correlation between the voice pressure and the

skin acceleration 22.2 g Pa^{-1}, was empirically obtained from the analysis of neck skin vibration for voice measurement.

Supplementary Note 2: Mechanical natural frequency of the diaphragm structure

The natural frequency of the diaphragm structure can be expressed as

$$f_0 = \frac{1}{2\pi}\sqrt{\frac{k}{m}} \cdots (2).$$

For the diaphragm structure with D = 400 μm, the mass of the diaphragm can be estimated as

$$m_{\text{dia.}} = A_{\text{dia.}} (\rho_{\text{Au}} t_{\text{Au}} + \rho_{\text{Ti}} t_{\text{Ti}} + \rho_{\text{SU-8}} t_{\text{SU-8}}) \cdots (3),$$

where $A_{\text{dia.}}$= 9.30 x 10^{-8} m^2 is the diaphragm area, ρ_{Au} = 19,300 kg m^{-3}, ρ_{Ti} = 4,506 kg m^{-3} and $\rho_{\text{SU-8}}$ = 1,200 kg m^{-3} are the densities of the components, and t_{Au} = 35 nm, t_{Ti} = 10 nm and $t_{\text{Su-8}}$ = 360 nm are their thickness. The mass of the diaphragm is 1.16 x 10^{-10} kg, The stiffness of the suspended diaphragm with air holes is 37.71 N m^{-1} (Supplementary Fig. 5). Thus, f_0 was calculated as 94,405 Hz, which is comparable to the simulated value of $f_{0,\text{simul.}} \approx$ 86 kHz (Supplementary Fig. 6).

Supplementary Note 3: Calculation for the residual stress of the diaphragm

According to the load-deflection model for a circular diaphragm[2], the relationship between applied pressure and diaphragm deflection is obtained as

$$P = \frac{4\,\sigma_0 h}{R^2}\omega_{\mathrm{d}} + \frac{16\,Eh^3}{3R^4(1-\nu^2)}\omega_{\mathrm{d}} + \frac{256\,Eh}{105R^4(1-\nu^2)}{\omega_{\mathrm{d}}}^3 \cdots \ (4),$$

where P [Pa] is the applied pressure, and σ_0 [Pa], h [m], R [m], ω_{d} [m], E [Pa] and ν represent residual stress, thickness, radius, center deflection, Young's modulus and Poisson ratio of the suspended diaphragm, respectively. In order, the three terms on the righthand side quantify how the residual stress, bending moments, and stress due to nonlinear stretching affect the stiffness of the diaphragm. For a diaphragm structure with thickness $\leq$500 nm and a radius $\geq$200 μm, the first term is three orders of magnitude higher than the second and third terms because the radius is much larger than the thickness and the center deflection. Therefore, Supplementary Equation (4) can be reduced to

$$P \approx \frac{4\,\sigma_0 h}{R^2}\omega_{\mathrm{d}} \cdots (5).$$

Multiplying P by the membrane area ($= \pi R^2$) yields the applied force

$$F\ = 4\pi\sigma_0 h\omega_{\mathrm{d}} \cdots (6).$$

For diaphragms that have air holes, Supplementary Equation (6) must be adjusted to include the reduced stiffness, compared to the diaphragms without holes. We applied the stiffness calibration constant $\kappa = 0.84$ obtained by comparing the simulated stiffness values of the diaphragms with and without air holes (Supplementary Fig. 5):

$$F\ = 4\kappa\pi\sigma_0 h\omega_{\mathrm{d}} \cdots (7).$$

Therefore, we can extract the residual stress of diaphragm with air holes to be

$$\sigma_0 = \frac{F}{4\kappa\pi h\omega_{\mathrm{d}}} = \frac{k_{\mathrm{measure}}}{4\kappa\pi h}\ \cdots (8),$$

where k_{measure} [N m^{-1}] is the measured stiffness of the hole-patterned diaphragm. Supplementary Equation (8) indicates a diaphragm with thickness of 400 nm and stiffness of 37.71 N m^{-1} (Supplementary Fig. 5) has a residual stress of 8.93 MPa.

Supplementary Note 4: Optimization of the diaphragm structural parameters

To increase the vibrational sensitivity S_{vib}, we optimized the structural parameters of diaphragm diameter D (200, 400 and 800 μm), diaphragm thickness t_D (400, 800 and 1200 nm), support thickness t_S (1.2, 1.6 and 2.0 μm) and the number of diaphragms (Figure 2b,c).

According to the load-deflection theory (see Supplementary Equation (6)), the stiffnesses of the diaphragms are the same at all D (Supplementary Fig. 9e). The diaphragm weight increases in proportion to D^2 and widens the change of the distance (Δd_{cap}) between the top and bottom electrodes (see second term of the righthand in Supplementary Equation (1)). In addition, the ratio of capacitance modulation to the change of the air gap under the diaphragm ($\Delta C/\Delta d_{cap}$) increases in proportion to D^2 (Supplementary Fig. 9d), so S_{vib} increases. However, as D increases, downward deflection of the diaphragm increases at a given operating DC voltage (3 V), so the thickness of the air gap under the diaphragm is reduced and the resistive force of the air gap is increased[3]. This phenomenon strongly impedes the improvement of S_{vib} at D = 800 μm, because the diaphragm is deflected ≥ 100 nm (Supplementary Fig. 9c). Therefore, as D increases, S_{vib} increases but dS_{vib}/dD declines. As mentioned later, D for the highest S_{vib} was determined after considering both the S_{vib} of a diaphragm, and the maximum number of diaphragms that can be fabricated in the same area.

The stiffness of the diaphragm increases with increasing t_D (Supplementary Note 3 and Supplementary Fig. 10e) and has a dominant effect on S_{vib} degradation. As t_D increases, the weight of the diaphragm also increases, but the increasing ratio is insignificant compared to the increase in diaphragm stiffness. This is because Au electrode on the diaphragm, sixteen times denser than SU-8, already contributes a large portion of diaphragm weight. $\Delta C/\Delta d_{cap}$ decreases slightly with increasing t_D, due to decrease of initial capacitance of SU-8 diaphragm. However, the capacitance change of the SU-8 diaphragm has a relatively small effect on the total capacitance, because the capacitance of the air-gap below the diaphragm, which is several times smaller than the capacitance of SU-8, has large effects on the total capacitance (Supplementary Fig. 10d). Therefore, as t_D increases, S_{vib} decreases.

t_S does not change the geometry of the diaphragm, so its stiffness and weight remain unchanged (Supplementary Fig. 11e). However, increase in t_S increases the thickness of the air film under the

diaphragm and decreases $\Delta C/\Delta d_{cap}$ by greatly decreasing initial capacitance (Supplementary Fig. 11d), so S_{vib} decreases. In contrast, increase in t_S reduces the resistive force of the air film and helps the diaphragm to deflect dynamically when the relative magnitude of increased distance to initial t_S is large. To summarize, as t_S increases, S_{vib} decreases and the magnitude of dS_{vib}/dt_S increases.

The diaphragm arrays are electrically connected in parallel, so S_{vib} is proportional to the number of diaphragms. However, the area of the device that can be attached to the neck skin to detect skin vibration is limited. The maximum number of diaphragms that can be fabricated in the same area is inversely proportional to D^2. For example, in the same area of 1 cm^2, we could fabricate 400, 100 or 25 arrays with diaphragms of D = 200, 400 or 800 μm, respectively (Supplementary Fig. 8). An array of diaphragm with D = 400 μm had slightly higher S_{vib} than did arrays with D = 800 μm and D = 200 μm. D has a greater effect than the number of arrays, but is constrained in the array of diaphragms that have D = 800 μm due to the resistive force of air film. Therefore, to maximize S_{vib}, D was determined to 400 μm.

Supplementary Note 5: Analysis on the attached position of the device for voice-recognition

The magnitude of neck skin vibration depends on the location of the skin during human speech; we analyzed the attached position of the device to obtain the best voice-recognition sensitivity. Two places of the skin near the cricoid cartilage and the thyrohyoid muscle were selected as candidates based on a previous study[4] which suggested that the two places tend to have a large magnitude of skin vibration. We measured the neck skin vibration by contactless laser Doppler vibrometer to eliminate mechanical interference with the vibration signal. The experimental results showed that the magnitude of acceleration on the neck skin was greater near the cricoid cartilage than near the thyrohyoid muscle (Supplementary Fig. 16).

Supplementary Note 6: Voice acoustic parameters

We adopt the definitions of voice acoustic parameters in Titze et al. (2003)[5] as follows. The phonation time (D_t) for speaking is defined as

$$D_\mathrm{t} = \int_0^{t_\mathrm{p}} 10 k_\mathrm{v} dt \ \ [\mathrm{s}] \cdots (9),$$

where k_v is the unit-step function for speaking divided into 100-ms intervals for performance time (t_p) :

$$k_\mathrm{v} = \begin{cases} 1 & for\ speaking \\ 0 & for\ non-speaking \end{cases} \cdots (10).$$

The total distance (D_d) of the vocal fold vibration can be defined as

$$D_\mathrm{d} = \int_0^{t_\mathrm{p}} 0.4 k_\mathrm{v} A F_0 dt \ \ [\mathrm{m}] \cdots (11),$$

where F_0 [Hz] is the fundamental voice frequency, and A [m] is vibration amplitude which can be empirically defined as

$$A \ = \ 0.05 L_0 \left(\frac{10^{\frac{(dB_{\mathrm{SPL}} - 72.5)}{27.3}}}{0.14 + 0.06 \left(\frac{F_0}{F_{0\mathrm{N}}} \right)^2} \right)^{\frac{1}{2}} \cdots (12),$$

where L_0 is the reference vocal fold length (16 mm for males; 10 mm for females), $\mathrm{dB_{SPL}}$ is the sound pressure of the voice at 1 m from the mouth and $F_{0\mathrm{N}}$ is a nominal fundamental frequency (120 Hz for males; 190 Hz for females)[6,7].

Energy dissipated (D_e) due to vibration of the vocal folds is defined as

$$D_\mathrm{e} = \frac{1}{20} \int_0^{t_\mathrm{p}} k_\mathrm{v} \eta \left(\frac{A}{T} \right)^2 \omega^2 dt \ \ [\mathrm{J\ m^{-3}}] \ \cdots (13),$$

where ω [rad $\mathrm{s^{-1}}$] is angular frequency, η [Pa s] is the shear viscosity which is empirically defined[S8] as

$$\eta = \frac{k_{\mathrm{h}}}{F_0} \cdots (14)$$

where k_{h} [Pa] is 5.4 for males and 1.4 for females, and T [mm] is the vocal fold thicknes[9-13] which is empirically related to F_0 as

$$T = \left(\frac{15.8}{1 + 2.15 \left(\frac{F_0}{F_{0\mathrm{N}}} \right)^{\frac{1}{2}}} \right) \text{ for males} \cdots (15)$$

and

$$T = \left(\frac{10.63}{1 + 1.69 \left(\frac{F_0}{F_{0\mathrm{N}}} \right)^{\frac{1}{2}}} \right) \text{ for females.} \cdots (16).$$

The temperature increase [°C] due to the dissipated energy can be obtained as

$$\Delta T = \frac{D_{\mathrm{e}}}{c\rho}, \cdots (17)$$

where c = 3,470 J $(\mathrm{kg\,C})^{-1}$ for human tissue is the specific heat capacity and ρ = 1,020 kg m^{-3} is the density of the tissues[14].

Supplementary Note 7: Electrostatic force applied to the diaphragm

To obtain the stiffness of the diaphragm (Supplementary Figs. 9e, 10e and 11e), we calculated the electrostatic force (F_e) applied to diaphragm under a DC voltage bias (V_bias) as

$$F_\mathrm{e} = \frac{1}{2}\frac{\partial C}{\partial W}{V_\mathrm{bias}}^2 \quad \cdots (18),$$

where $\frac{\partial C}{\partial W}$ is the change of capacitance (C) due to the deflection (W) and was obtained as the slope of the measured data (Supplementary Figs. 9d, 10d and 11d).

■ Supplementary References

1 Younis, M. I. *MEMS linear and nonlinear statics and dynamics*. *Vol. 20* (Springer Science & Business Media, 2011).

2 Schomburg, W. K. *Introduction to Microsystem Design* (Springer, 2011).

3 Warren, J. *et al.* Capacitance microphone dynamic membrane deflections. *J. Acoust. Soc. Am.* **54**, 1201-1213 (1973).

4 Nolan, M. *et al.* Accelerometer based measurement for the mapping of neck surface vibrations during vocalized speech. *31st Conf. Proc. IEEE Eng. Med. Biol. Soc.* 4453-4456 (2009).

5 Titze, I. R. *et al.* Vocal dose measures: quantifying accumulated vibration exposure in vocal fold tissues. *J. Speech Lang. Hear. Res.* **46**, 919-932 (2003).

6 Titze, I. R. Phonation threshold pressure: a missing link in glottal aerodynamics. *J. Acoust. Soc. Am.* **91**, 2926-2935 (1992).

7 Titze, I. R. & Sundberg, J. Vocal intensity in speakers and singers. *J. Acoust. Soc. Am.* **91**, 2936-2946 (1992).

8 Chan, R. W. & Titze, I. R. Viscoelastic shear properties of human vocal fold mucosa: measurement methodology and empirical results. *J. Acoust. Soc. Am.* **106**, 2008-2021 (1999).

9 Hollien, H. Vocal fold thickness and fundamental frequency of phonation, *J. Speech Lang. Hear. Res.* **5**, 237-243 (1962)

10 Hollien, H. & Colton, R. Four laminagraphic studies of vocal fold thickness. *Folia Phoniatrica et Logopaedica* **21**, 179-198 (1969).

11 Hollien, H. & Curtis, J. F. A laminagraphic study of vocal pitch. *J. Speech Lang. Hear. Res.* **3**, 361-371 (1960).

12 Hollien, H. & Moore, G. P. Measurements of the vocal folds during changes in pitch. *J. Speech Lang. Hear. Res.* **3**, 157-165 (1960).

13 Nishizawa, N. *et al. Vocal Physiology: voice production, mechanisms and functions* (Lippincott Williams & Wilkins, 1988).

14 Perlman, A. L. & Titze, I. R. Development of an in vitro technique for measuring elastic properties of vocal fold tissue. *J. Speech Lang. Hear. Res.* **31**, 288-298 (1988).
