# Supplementary material for: An ultrathin conformable vibration-responsive electronic skin for quantitative vocal recognition
Source: Nat Commun. 2019 Jun 18;10:2468. doi: 10.1038/s41467-019-10465-w (PMC6581939; doi:10.1038/s41467-019-10465-w)
Supplement: Supplementary file 3 — Description of Additional Supplementary Files [file 41467_2019_10465_MOESM3_ESM.pdf]

Description of Additional Supplementary File

File Name: Supplementary Movie 1

Description: Demonstration of voice authentication system and voice remote control system by connecting our device to a speech-recognition module. The demonstration was performed for three cases as follows.

1) Administrator in a normal acoustic environment

2) Unauthorized user in a normal acoustic environment

3) Administrator wearing a mask
